# Heterogeneous Influences of Social Support on Physical and Mental Health: Evidence from China

**DOI:** 10.3390/ijerph17186838

**Published:** 2020-09-18

**Authors:** Fan Yang, Yao Jiang

**Affiliations:** 1 Department of Labor and Social Security, School of Public Administration, Sichuan University, Chengdu 610065, China; 2 Department of Accounting, School of Management, Sichuan Agricultural University, Chengdu 611130, China; s20175706@stu.sicau.edu.cn

**Keywords:** social support, physical health, mental health, heterogeneity, China

## Abstract

Employing a national representative survey (the China Labor-force Dynamics Survey 2016, CLDS2016) data (*N* = 14246), this paper examines the heterogeneous influences of social support on individual physical and mental health in China. Social support is characterized by four dimensions: emotional support, tangible or instrumental support, interaction or exchange support, and community support. Physical health is measured by self-rated health and body mass index (BMI), while mental health is measured by depression, hopelessness, failure, fear, loneliness, and meaninglessness. The results indicate that different dimensions of social support have heterogeneous effects on individual physical and mental health. Specifically, the correlation between emotional support and individual physical health is not significant, but emotional support is significantly related to some mental health variables. Tangible or instrumental support is significantly related to individual self-rated physical health but not to BMI or mental health. Interaction or exchange support is significantly correlated with individual self-rated health and some mental health variables. In general, there are significant correlations between community support, and individual physical and mental health. The results also suggest that the influences of social support on physical and mental health of individuals at different ages (<60 years and ≥60 years) are heterogeneous. The results of this study provide direction for the dimension selection of social support to promote individual health.

## 1. Introduction

Individuals are embedded within a society, and social support affects multitudinous aspects of individuals, including health. Individual good health is a valuable aspect of life and social development, and the relationship between social support and individual health is receiving increased academic attention [1,2,3]. While social support can affect both individual physical and mental health [4], it is unclear which is more closely related to social support. This paper investigates whether there is any heterogeneity in the effects of multidimensional social support on physical and mental health. Further, the influences of multidimensional social support on health at various ages are investigated. Answers to the above issues have not reached academic consensus, as evidenced by a literature review conducted in this study.

Social support refers to the care and support that social members can receive from others [5]. It can improve individual social adaptability [6] and is a potential social factor affecting individual health [7]. As early as the 1940s, the World Health Organization (WHO) presented a multidimensional definition of health, which was a state of complete physical, mental, and social well-being and not merely the absence of disease or infirmity [8]. As can be seen from this definition, health is a multidimensional concept, including not only physical health but also mental health. From 1990 to 2016, the burden of mental disorders globally was enormous, with an estimated 1.1 billion population affected by mental or substance use disorders [9]. A growing body of literature has demonstrated that the amount and quality of social support from relatives, friends, neighbors, and the community are pivotal factors in positively affecting a person’s physical and mental health [10,11,12] and acts as a form of prevention against harmful behaviors and distressing emotions [13].

Social support is one of the well-documented factors influencing physical health outcomes [14,15]. The most compelling evidence on the physical health outcomes of social support is a meta-analysis of the existing literatures that found that social support significantly lowers the risk for mortality [16]. Studies have shown that, the stronger a person’s social support network, the more likely they are to obtain more wealth, higher social status, as well as medical resources to prevent diseases and to maintain good physical health [17,18]. These individuals with strong social support networks can use these supports to receive good treatment when faced with disease [19]. Conversely, those who do not have much social support may not have enough resources to remain healthy. When they are suffering from disease, it is also challenging for them to obtain good medical resources or to pay for treatment, which causes their health to further deteriorate [19].

Previous studies have argued that social support is a factor affecting mental health, and these studies have shown that there are two primary ways that social support affects individual mental health [20,21]. The first is main (or additive) effects of social support on health [22]. It is argued that social support has a generally beneficial effect. When the amount of social support increases, the level of individual mental health improves [22]. The second is stress-buffering (also termed moderating or interactive) effects of social support on mental health [23]. In this case, research suggests that social support only plays a role in mental health under stress. Social support minimizes the impacts of stress from negative life events on psychological health [24].

However, empirical studies do not reach a consensus on the two ways that social support affects individual mental health. Some studies have found evidence of the main or stress-buffering effect of social support on health [25,26,27,28]. For example, a study on the mental health of incarcerated offenders showed that perceived social support helps safeguard the mental health of offenders [29]. Further, a study of college students with disabilities showed that both the main and buffering effects of social support effectively relieve their financial pressure [30]. Conversely, other studies have shown that the effect of social support on mental health is not significant [31,32]. For example, a five-year longitudinal study has found that social support does not uniformly mitigate the effects of stressors on health for individuals living in urban poverty [33].

In conclusion, previous researchers have mainly studied the correlations between social support and health from the two perspectives of physical and mental health [34,35]. These two perspectives should be compared, but the results of the presented works may be incomplete if analyzed from one perspective alone.

Social supports are multifaced [36,37]. Previous studies mainly used functional and global functional concepts to measure it. Functional social support refers to the functions performed for individuals by significant others or secondary group members. The most frequently noted functions are emotional, informational, and instrumental assistance [36]. The measurement of global functional social support combines the functional social supports mentioned above into a single index [38]. In this paper, under the consensus that total social support influences individual health, we do not intend to combine varying functional social supports into a single index. Instead, we investigate the heterogeneous influences of various functional social supports on individual physical and mental health.

Based on previous studies, we divided social support into four dimensions: emotional support, tangible or instrumental support, interaction or exchange support, and community support [39,40]. Emotional support plays a protective role in individual physical and mental health. For physical health, studies have demonstrated that individuals who lack emotional support are twice as likely to commit suicide and to suffer from myocardial death and cardiac disease compared to individuals who have emotional support [41,42]. For mental health, emotional support is associated with a reduction in psychological distress and anxiety [43,44]. Tangible or instrumental support refers to giving individuals practical support, such as financial assistance [45]. Effective tangible or instrumental support can help individuals maintain their general health or recover from illness [22]. From the perspective of psychology, productive interaction or exchange support involves having people who can discuss important personal issues. Not only does this release individual anxiety and pressure but also this kind of support enables collaborative solutions to be reached. [46]. In China, with the improvement of rural and urban community management, the grassroots community plays an increasingly vital role in people’s lives, which includes their health [47]. Members of a community often form an intimate group. Through mutual acquaintance, trust-building, and mutual assistance, they can become a source of social support for each other [10].

In terms of specific measurement indicators, the number of friends a person has is used to measure emotional support [48]. Tangible or instrumental support is commonly measured by the number of people that an individual can borrow money from [49,50]. Interaction or exchange support is usually measured by the number of people who can discuss important personal issues together [51]. Community members’ familiarity, trust, and mutual assistance is used to measure the level of community support [52].

In addition to social support, individual health is also affected by other factors. Gender, age, religion, marital status, health habits, and socioeconomic status are individual characteristics that are intimately related to health [53]. Gender is a widely documented determinant of health. Studies have indicated that feminine and undifferentiated gender roles are related to poor self-rated health and that the average health status of men is better than that of women [54]. It is recognized that individual health declines with age [55,56]. Regarding an individual’s marital status, single people experience higher mortality and poorer health than married people [57]. Studies on the link between religion and mental health have consistently revealed that spiritual people turn to their religious beliefs as one of the first resources when faced with traumatic life events or significant stressors [58,59]. Multiple studies of the factors influencing individual health have found compelling evidence concerning damaging health habits and behaviors such as smoking, drinking, and beneficial habits like regular physical activity [60]. An individual’s socioeconomic position involves the indicators of income, occupational prestige, and attaining education, which are intimately linked to health care accessibility and health literacy [61,62]. Studies have consistently documented that individuals with high incomes and good education are healthier than poorer, less educated people [63,64,65].

Apart from individual characteristics, the environment in which the person is located also influences individual health. A growing, global body of literature has focused on the negative impacts of environmental pollution, especially air pollution, on individual health [66,67,68,69,70,71].

Studying the health of Chinese people has great social significance for both the general public and for the Chinese government. First, according to data released by the Chinese government, about 40% of the poverty experienced by China’s rural population is caused by health problems [72]. This means that, with increased social support, improving people’s health can play a role in alleviating poverty. Second, there was a large seasonal and internal migrant population in China, consisting of around 250 million people at the end of 2019 [73]. They migrated from rural areas where their household was registered to urban places to seek work. In this process, whether social support is available may affect individual income as well as health. Additionally, the first Blue Book of Chinese Mental Health (2017–2018), released by the Chinese Academy of Sciences Institute of Psychology, shows that an increasing number of individuals experience psychological problems in China [74]. The Chinese government is committed to building a healthy population in China. Citizens’ health is a symbol of national prosperity. The government is gradually improving national health policies to provide people with comprehensive health services. Improving individual social support is part of these policies. In conclusion, it is of great pragmatic significance to study the effect of social support on the health of Chinese people.

This paper aims to fill some of the gaps in current studies on individual health. Based on big data from China, this study adopts quantitative research methods to analyze the heterogenous affecting of social support on individual physical and mental health. First, this study attempts to give a description of the influence of multidimensional social support on both individual physical and mental health. Second, this study shows a comparison of each dimension of social support on individual physical and mental health, which strongly proves the varied effects of social support on health. Finally, this study expands the heterogeneity to age and effectively identifies the heterogeneous influence of each dimension of social support on different ages’ mental health.

## 2. Methods

### 2.1. Survey Designs

The data of this paper comes from the China Labor-force Dynamics Survey 2016 (CLDS2016) carried out by Sun Yat-sen University in 2016. The survey covers education, work, migration, health, economic activities, and other interdisciplinary aspects. In this survey, a multi-stage, multi-level probability sampling method proportional to the size of the labor force is adopted. To ensure national representation, the samples cover 29 provincial administrative units (Hong Kong, Macao, Taiwan, Tibet, and Hainan are not included). Therefore, this dataset is highly representative of China. It is a public dataset that all researchers can use by applying to Sun Yat-sen University. The survey is conducted by Computer Assisted Personal Interviewing (CAPI) technology. In order to reduce the estimation bias as much as possible, this paper removes invalid samples in the original data table. Specifically, the samples with the following characteristics have been deleted: refusing to answer key questions or answering “inapplicable, unclear” and obvious logical contradictions. Finally, 14,246 valid samples were used in this paper. Therefore, the data this paper employed can be regarded as big data in terms of both the national representation of the survey scope and the absolute number.

### 2.2. Measurements

#### 2.2.1. Dependent Variables

Two dimensions, physical health and mental health, of respondents were measured. For the first dimension, physical health, survey participants were asked, “How do you evaluate your current health (variable named self-rated health)?” The answer was measured using a five-point Likert scale ranging from “1” to “5”. An answer of “very bad” was coded as “1”, “bad” was coded as “2”, “normal” was coded as “3”, “good” was coded as “4”, and “very good” was coded as “5”. Considering the subjectivity of self-rated health, this paper also used body mass index (BMI) to measure the physical health of respondents. In the standards provided by the World Health Organization (WHO), 18.5 ≤ BMI < 24 refers to a normal weight range [75]. However, according to a study published in the Lancet by WHO experts, a normal BMI between 18.5–23 may be more appropriate for Asians [76]. Therefore, we chose the range of BMI from 18.5 to 23 as normal weight. If 18.5 ≤ BMI < 23, it was coded as “1”; otherwise, it was coded as “0”.

Individuals who develop mental health problems may experience feelings of depression, hopelessness, failure, fear, loneliness, and meaninglessness [77]. Therefore, for the second dimension, mental health, respondents were asked six questions: “How often do you feel depressed (variable named depression)?”, “How often do you feel like there is no hope (variable named hopelessness)?”, “How often do you feel you have failed (variable named failure)?”, “How often do you experience fear (variable named fear)?”, “How often do you feel lonely (variable named loneliness)?”, and “How often do you feel life is meaningless (variable named meaninglessness)?” The answers were coded from “1” (very low frequency) to “4” (very high frequency), which meant that the individual mental health status was ranked from good to poor [78,79].

#### 2.2.2. Explanatory Variables

Explanatory variables in social support include four dimensions: emotional support, tangible or instrumental support, interaction or exchange of support, and community support. For the first dimension, emotional support, respondents were asked two questions: “How many friends do you have locally (variable named friends)?” and “How many people can you speak your mind to (variable named speaking one’s mind)?” The answers to both questions were numerical. In other words, emotional support was defined by two variables: friends and speaking one’s mind.

For the second dimension, tangible or instrumental support, respondents were asked, “How many people can you borrow money from (variable named borrowing money)?” The answer to the question was also numerical. It meant that tangible or instrumental support was defined by the variable of borrowing money.

For the third dimension, interaction or exchange of support, respondents were asked, “How many people can you discuss important personal issues with (variable named discussion)?” The answer was still numerical. In other words, interaction or exchange of support was defined by the variable of discussion.

For the fourth dimension, community support, respondents were asked three questions: “How familiar are you with the members in your community (variable named familiarity)?” The answer was measured by a five-point Likert scale ranging from “1” (very unfamiliar) to “5” (very familiar). “To what extent do you trust the members in your community (variable named trust)?” The answer was also measured using a five-point Likert scale ranging from “1” (very distrustful) to “5” (very trustful). The final question of the fourth dimension was “Do you have mutual aid with the members in your community (variable named mutual aid)?” The answer was again measured by a five-point Likert scale ranging from “1” (very little) to “5” (very much). It meant that community support was defined by three variables: familiarity, trust, and mutual aid.

#### 2.2.3. Control Variables

According to the analysis in the introduction section, the control variables of this paper included gender, age, education, marital status, religion, income, working time, smoking, drinking, exercise, and region. Gender was a dummy variable. Male was coded as “1”, and female as “0”. Age was a continuous variable. Education referred to the number of years of schooling, which was also a continuous variable. Marital status was a dummy variable, which was divided into “single”, “married”, “divorced”, and “widowed”. Religion was a dummy variable, which was clustered into “Western religion (including Catholicism, Christianity, and the Eastern Orthodox Church)”, “Eastern religion (including Southern Buddhism, Tibetan Buddhism, Taoism, Islam, and folk religions)”, and “no religion”. Income was a continuous variable, which referred to the total income of respondents in 2015, mainly composed of wage income, operating income, property income, and transfer income. In regression analysis, we took the logarithm of income. Working time was measured by the average number of days respondents worked in one month ranging from “0” to “31”. Smoking and drinking both were dummy variables with “1” representing “yes” for each of the two variables. Exercise was measured by asking the question, “Do you exercise regularly in your daily life?”, with “1” representing “yes”. Region was a dummy variable and was measured by the provinces where respondents were located.

### 2.3. Data Analysis Strategy

The eight measures of outcome, (1) self-rated health, (2) BMI, (3) depression, (4) hopelessness, (5) failure, (6) fear, (7) loneliness, and (8) meaninglessness, were used as the dependent variables. The ordered probit (oprobit) regression models were used to estimate the results of (1) self-rated health, (3) depression, (4) hopelessness, (5) failure, (6) fear, (7) loneliness, and (8) meaninglessness, due to these dependent variables being ordered discrete data. The logistic regression model was used to estimate the result of (2) BMI, due to the BMI being measured as binary. The statistical software Stata version 13.1 MP was used to implement the analysis (StataCorp. LP., College Station, TX, USA).

There are two main limitations of this paper. One is in research and design. Our data is second-hand data collected by other research institutions. Health and social support are only part of this dataset. In addition, mental health is a complex concept, so it is extremely difficult to quantify accurately. Therefore, we only use six indicators to measure mental health, which is obviously not enough to represent its complexities. Future research can cautiously expand the dimensions of mental health. The second limitation of this paper is in the methods used. This is also related to the data. Due to the cross-sectional nature of the data, this paper does not explore the internal mechanisms of social support for physical and mental health. Future research can continue to expand on this point.

## 3. Results

### 3.1. Descriptive Analysis

Table 1 shows the distribution of the samples: 14,246 subjects were included in the samples. Out of the total number, 53.66% of the subjects were male while 46.34% were female. The ages of all samples ranged from 20 to 91 years old, with an average age of 50.64 (SD = 12.99) years. The average years of education for respondents were 8.68 years, with a minimum of 0 (meaning illiteracy) and a maximum of 23 (meaning doctoral degree). The average income of respondents was 31,956.91 yuan in 2015. The average work time of respondents was 22.92 days a month. The marital status of respondents showed that 9.67% were single, 86.73% were married, and 1.54% were divorced and that the last 2.06% are widowed. Of the respondents, 32.31% smoked and 23.57% drank; 28.32% engaged in regular exercise; and 2.34% were part of Western religions, 10.17% held Eastern religious beliefs, and the other 87.49% had no religious beliefs.

In terms of physical health, the average value of self-rated health is 3.65 (SD = 0.97). BMI shows that 46.1% of the respondents’ weights were within the normal range. In terms of mental health, the mean values of six indicators are all less than 2, among which, the mean value of depression is 1.33 (SD = 0.59), the mean value of hopelessness is 1.34 (SD = 0.67), the mean value of failure is 1.31 (SD = 0.63), the mean value of fear is 1.23 (SD = 0.52), the mean value of loneliness is 1.29 (SD = 0.60), and the mean value of meaninglessness is 1.24 (SD = 0.55).

When reviewing social support factors that may influence physical and mental health, on average, respondents had 12.33 friends, 4.88 respondents had people with whom they could speak their mind, 5.08 people from whom they could borrow money, and 4.01 people with whom they could discuss important personal issues. In terms of community support, the average degree of familiarity of the respondents and other members of the community is 3.81, the average degree of trust of the respondents and the other members of the community is 3.68, and the average value of mutual aid between the respondents and the other members of the community is 3.36.

### 3.2. Influence of Social Support on Physical Health

The influences of the social support factors on the two physical health dimensions, self-rated health and BMI, are estimated separately by an oprobit regression model and a logistic regression model. The results are shown in Table 2. The number of samples used in the estimations is 14,246. Varied technical diagnostic tests were conducted [80,81], and the results show that the two models are good fits.

It can be observed from Table 2 that the influences of social support on self-rated health and BMI are heterogeneous. Specifically, the two indicators of emotional support (friends and speaking one’s mind) do not significantly affect self-rated health and BMI. Tangible or instrumental support (borrowing money) significantly and positively affects self-rated health but not BMI. This result means that, with the increase of tangible or instrumental support (borrowing money), individual self-rated health level is correspondingly higher on average.

Similarly, interaction or exchange support (discussion) affects self-rated health significantly and positively but not BMI. On average, individuals with more people to discuss important personal issues with have higher self-rated health. In terms of community support, the respondents’ degree of familiarity with other members of the community has a significant and positive effect on the self-rated health of the respondents and has a significant and negative effect on BMI. The degree of trust that the respondents have with the other members in their community has a significant positive effect on both self-rated health and BMI. The frequency of mutual aid behavior of the respondents and community members significantly and positively affects the self-rated health of the respondents but not their BMI.

For the results of the control variables, on average, the older the respondent is, the lower their self-rated health level is. Similarly, the older the respondent is, the lower the probability that their weight is within the normal range. For education, the more years of schooling the respondent has, the higher their self-rated health level is. In terms of income, the self-rated health level of the respondent increases with the increment of annual income. Finally, people who adhere to regular exercise have higher self-rated health levels compared with those who do not exercise regularly.

### 3.3. Influence of Social Support on Mental Health

Oprobit regression models are used to estimate the influences of social support factors on the six mental health dimensions in this study (depression, hopelessness, failure, fear, loneliness, and meaninglessness). The results are shown in Table 3. The number of samples used in the estimations is also 14,246, and the models are found to be a good fit [80].

Table 3 shows that the more friends the respondents have, the higher frequency they feel depression, failure, and fear; the more people the respondent has to discuss important personal issues with, the less likely respondents will experience feelings of hopelessness, failure, and loneliness; and the more familiar the respondents are with members of their community, the better their mental health is, that is, the less likely the respondents will feel depressed, hopeless, afraid of failure, fearful, lonely, and meaningless. Similar results appeared as the respondents were more trusted within their community. The more mutual aid behaviors the respondents have with other members of their community, the better their mental health is, and respondents thus experience less frequent feelings of hopelessness, failure, loneliness, and meaninglessness.

In the control variables, on average, compared with women, men’s mental health is better. They spend less time feeling depressed, hopeless, like a failure, fearful, lonely, and meaningless than women do. The amount of time that respondents feel these variables increases with age. The higher the level of education of the respondents, the lower the frequency of depression, hopelessness, failure, fear, loneliness, and meaninglessness they feel. Regarding marital status, compared with single people, married respondents have better mental health. They spend less time feeling the above variables than single people do. The more income the respondents earn, the less likely they are to feel depressed, hopeless, failed, feared, lonely, and meaningless. Respondents who exercise regularly are also less likely to feel these variables.

### 3.4. Influence of Social Support on Mental Health of Different Ages

Age is significantly associated with mental health [82]. As such, we grouped the samples into two subgroups—respondents below 60 and those 60 and over—to check the heterogeneous influence of emotional, tangible or instrumental, interaction or exchange, and community support on individual mental health at different ages. The results are reported in Table 4.

It can be observed from Table 4 that not all the variables (friends and speaking one’s mind) related to emotional support have significant effects on individual mental health across the two different age subgroups. Specifically, the number of friends that respondents have has significant negative effects on the mental health variables of depression, failure, and fear in the below-60 subgroup. The variable of speaking one’s mind shows a significant positive effect on easing the feeling of failure in the 60-and-over subgroup, while it has no significant effects on the other mental health variables.

For tangible or instrumental support, the variable of borrowing money only has a significant positive effect of relieving feelings of depression, hopelessness, and loneliness in the 60-and-over subgroup. The variable did not affect the mental health of those aged below 60.

In terms of interaction or exchange support, the coefficients of variables of discussion are insignificant across the two subgroups, demonstrating that the interaction or exchange of support does not affect respondents’ mental health status positively or negatively.

Overall, community support is the most crucial dimension of social support affecting individual mental health. The degree of familiarity respondents share with the other members of their community has significant, positive influences on every assessed mental health status in the below-60 subgroup. In contrast, in the 60-and-over subgroup, the degree of familiarity lessens the feelings of depression and fear significantly. The degree of trust that the respondents share with the other community members improves overall mental health across the two different subgroups significantly. Alternatively, the trust between community members shows that there is a strong association of mental health with community support among individuals. The mutual aid behaviors of the respondents and other community members is significantly and positively correlated with alleviating feelings of hopelessness, failure, and meaninglessness but significantly exacerbates the feeling of fear in the 60-and-over subgroup. For the below-60 subgroup, the effects of mutual aid are significantly positive and help alleviate loneliness and meaninglessness.

## 4. Discussion

By analyzing updated and representative survey big data from China, this paper examines the influence of social support on individual health. Different from previous studies, we examine the influences of social support on individual physical and mental health and attempt to find heterogeneity between them.

We find that the number of friends that respondents have has no significant influence on their own physical health (self-rated health and BMI). However, the number of friends has a significant influence on some aspects of respondents’ mental health (depression, failure, and fear). We questioned why people with more friends feel more depressed, failed, and fearful. We speculate that this is related to the comparative effect. To a large extent, the psychological problems of individuals come from people close to them, such as family members and friends, rather than strangers [83,84]. The more friends a person has, the more people the person can compare themselves with. Generally, the more friends a person has, the greater the probability of having more highly accomplished friends. Comparing oneself with those talented friends may induce depression, a sense of failure, and fear. This is not the fault of the friends but instead how people think about the role of friends in their lives.

The number of people that the respondents could borrow money from significantly affects the respondents’ self-rated health but does not significantly affect BMI and mental health. Based on these results, we can argue that the influence of tangible or instrumental support on individual health, especially mental health, is limited. Therefore, these results above provide evidence for future studies to reconsider the heterogeneous effects of different dimensions of social support on individual health. Additionally, there is the potential for future studies to consider whether tangible or intangible support has the most significant effect on individual health.

Having more people with whom the respondent can discuss important personal issues not only can improve the self-rated health of respondents but also can make them feel less hopeless, failed, and lonely. The importance of discussion is reflected in that it can reduce the cognitive limitations of an individual, can open one’s mind, can find solutions to problems, and can create a sense of hope. Helping individuals find others to discuss things with will be a potential way that government and nongovernmental organizations (NGOs) can provide social support for individuals. Specifically, the government and NGOs can set up community-based advice agencies to provide constructive suggestions on the problems that individuals encounter in daily life to increase their social support.

The more familiarity the respondents have with other community members, the higher their self-rated health level is but the greater the probability that their BMI is within an abnormal range. Although it is difficult to give a reasonable explanation for the above results, it shows that different choices of health indicators may produce different results. Whether to choose subjective or objective indicators to measure physical health is worthy of further study. However, the influence of this variable (familiarity) on the mental health of respondents is consistent. It has a significant positive effect on the six variables (depression, hopelessness, failure, fear, loneliness, and meaninglessness) of mental health. The more familiar the respondents are with other community members, the better their mental health is and the less depressed, hopeless, failed, fearful, lonely, and meaningless they feel. This is evidence that community support has a significant effect on individual mental health, consistent with previous studies [85]. This result suggests that community involvement should be emphasized when strengthening social support. Specific measures may include holding communal activities for promoting fellowship, so that community residents can be well acquainted with each other.

There is a significant correlation between the degree of trust of respondents with the other community members and the respondents’ mental health. The more trust the respondents have with other community members, the better their mental health is. Trust is the foundation of mental health [86,87]. Against the social background of the trust crisis in China [88], this result reminds us again that we cannot ignore the role of trust in people’s mental health. Positive measures should be taken to maintain interpersonal trust, which is not only beneficial to the mental health of social members but also beneficial to the healthy development of the whole society.

Mutual aid behavior has a significant correlation with hopelessness, failure, loneliness, and meaninglessness. The more the members of a community help each other, the less time members experience hopelessness, a sense of failure, loneliness, and meaninglessness. However, the frequency of mutual aid behavior within a community significantly affects the self-rated health of respondents but not BMI. The results remind us again that, in the process of measuring health, choosing different indicators may produce different results. Additionally, the above results suggest that mutual aid behavior is beneficial to people’s mental health. Therefore, it is necessary for the community to establish a set of effective mechanisms to stimulate community members to help each other. To conclude, community support plays a prominent role in the four dimensions of social support.

Our study shows that age plays a moderating role in the impact of social support on mental health. People under the age of 60 and people aged 60 and above have heterogeneous perceptions about the effects of various social support dimensions on different mental health indicators. Therefore, the social support measures provided to these two age groups to help them improve their health must also be heterogeneous and targeted. Specifically, according to the results of this paper, community support is needed by both age groups (<60 and ≥60), tangible or instrumental support is needed more by the age group over 60 (including 60), and more emotional support should be given to the age group below 60.

For the control variables, the results are mostly consistent with previous studies. In particular, we find that the influence of gender on mental health is heterogeneous. On average, compared with women, men’s mental health is better. In addition to the psychological and physiological differences of gender, it may also be related to the different social division of labor between men and women in China. Although the status of women has been greatly improved in China, generally, most women are still in a subordinate position in a family. Household chores are dealt with mainly by women, which can easily lead to mental health issues [89]. Therefore, women’s mental health problems deserve further attention. Reasonable family division of labor may help to alleviate this problem. In addition, we find that marriage is a way to alleviate mental health problems. Compared with single people, those who are married enjoy better mental health. They spend less time feeling depressed, hopeless, like a failure, fearful, lonely, and meaninglessness than single people. Good interaction and communication between a husband and wife are beneficial to one’s mental health [90].

## 5. Conclusions

In conclusion, this paper has shown that different social support dimensions have heterogeneous effects on individual physical and mental health. Specifically, tangible or instrumental support (borrowing money), interaction or exchange support (discussion), and community support (familiarity, trust, and mutual aid) are significantly correlated with individual self-rated health. Community support (familiarity and trust) is significantly correlated with individual BMI. Compared with the other three dimensions, community support plays the most important role in individual mental health. This paper finds that friends do not play a positive role in the depression, failure, and fear dimensions of individual mental health. The results also suggest that the effects of social support on the physical and mental health of individuals at different ages (<60 years and ≥60years) are heterogeneous. In addition, this study reminds us that different health measurement methods may produce different results. Therefore, scientific measurement of health is the key to achieving more accurate results on this topic in future research. This paper has contributed to the literature on the heterogeneous influence of social support on individual physical and mental health in China.

## Figures and Tables

**Table 1:** Descriptive analysis (*N* = 14246).

**Variable**	**Mean**	**SD**	**Min**	**Max**
Self-rated health	3.65	0.97	1	5
Depression	1.33	0.59	1	4
Hopelessness	1.34	0.67	1	4
Failure	1.31	0.63	1	4
Fear	1.23	0.52	1	4
Loneliness	1.29	0.60	1	4
Meaninglessness	1.24	0.55	1	4
Friends	12.33	59.12	0	3000
Speaking one’s mind	4.88	31.67	0	3000
Borrowing money	5.08	77.61	0	5000
Discussion	4.01	17.10	0	1000
Familiarity	3.81	0.99	1	5
Trust	3.68	0.85	1	5
Mutual aid	3.36	1.03	1	5
Age (year)	50.64	12.99	20	91
Education (year)	8.68	4.26	0	23
Income (yuan)	31,956.91	57,822.36	0	3,050,000
Working time (day)	22.92	8.46	0	31
**Variable**	**Item**	**Freq.**		**Percent**
BMI	Normal	6567		46.10
Gender	Male	7645		53.66
Marital status	Single	1378		9.67
	Married	12355		86.73
	Divorced	220		1.54
	Widowed	293		2.06
Religion	Western	333		2.34
	Eastern	1449		10.17
	No religion	12,464		87.49
Smoking	Yes (“1”)	4603		32.31
Drinking	Yes (“1”)	3358		23.57
Exercise	Yes (“1”)	4035		28.32
Total		14246		100

**Table 2:** Influence of social support on physical health (*N* = 14246).

Variable	Self-Rated Health	BMI
	Oprobit	Logistic
Friends	0.0002	−0.0003
	(0.0002)	(0.0004)
Speaking one’s mind	0.0001	−0.0025
	(0.0004)	(0.0018)
Money borrowing	0.0002 *	0.0000
	(0.0001)	(0.0002)
Discussion	0.0011 *	0.0022
	(0.0006)	(0.0018)
Familiarity	0.0249 **	−0.0401 *
	(0.0122)	(0.0231)
Trust	0.0926 ***	0.0750 ***
	(0.0144)	(0.0274)
Mutual aid	0.0381 ***	−0.0140
	(0.0113)	(0.0215)
Gender (male)	0.0736 ***	−0.1573 ***
	(0.0243)	(0.0462)
Age	−0.0243 ***	−0.0069 ***
	(0.0009)	(0.0017)
Education	0.0221 ***	-0.0028
	(0.0027)	(0.0051)
Marital status (single as reference)		
Married	0.0052	−0.2541 ***
	(0.0349)	(0.0652)
Divorced	−0.0660	0.0197
	(0.0792)	(0.1498)
Widowed	−0.1045	−0.1483
	(0.0732)	(0.1393)
Religion (Western religions as reference)		
Eastern religions	0.0629	−0.1004
	(0.0676)	(0.1285)
No religion	0.0688	−0.0464
	(0.0604)	(0.1147)
Income	0.0418 ***	−0.0039
	(0.0038)	(0.0072)
Working time	0.0038 ***	0.0021
	(0.0011)	(0.0021)
Smoking (yes)	0.0011	0.1761 ***
	(0.0250)	(0.0476)
Drinking (yes)	0.0608 **	−0.0895 *
	(0.0241)	(0.0458)
Exercise (yes)	0.0985***	−0.2343 ***
	(0.0213)	(0.0404)
Region	Yes	Yes
Pseudo-R2	0.0703	0.0209

Notes: Standard errors in parentheses; *** *p* < 0.01, ** *p* < 0.05, * *p* < 0.1.

**Table 3:** Influence of social support on mental health (*N* = 14246).

Variable	Depression	Hopelessness	Failure	Fear	Loneliness	Meaninglessness
Friends	0.0004 **	0.0000	0.0006 ***	0.0006 ***	0.0001	−0.0002
	(0.0002)	(0.0002)	(0.0002)	(0.0002)	(0.0002)	(0.0003)
Speaking one’s mind	−0.0002	0.0004	−0.0012	−0.0012	−0.0012	−0.0006
	(0.0004)	(0.0004)	(0.0011)	(0.0011)	(0.0019)	(0.0015)
Borrowing money	−0.0001	−0.0002	−0.0001	−0.0024	−0.0002	−0.0001
	(0.0002)	(0.0002)	(0.0002)	(0.0016)	(0.0002)	(0.0002)
Discussion	−0.0007	−0.0035 **	−0.0034 *	−0.0014	−0.0044 *	−0.0010
	(0.0008)	(0.0017)	(0.0020)	(0.0017)	(0.0026)	(0.0018)
Familiarity	−0.0579 ***	−0.0475 ***	−0.0576 ***	−0.0796 ***	−0.0861 ***	−0.0674 ***
	(0.0148)	(0.0147)	(0.0150)	(0.0164)	(0.0152)	(0.0159)
Trust	−0.0666 ***	−0.0794 ***	−0.0878 ***	−0.0617 ***	−0.0610 ***	−0.0824 ***
	(0.0174)	(0.0174)	(0.0177)	(0.0194)	(0.0180)	(0.0187)
Mutual aid	0.0042	−0.0366 ***	−0.0305 **	0.0344 **	−0.0342 **	−0.0501 ***
	(0.0138)	(0.0138)	(0.0140)	(0.0154)	(0.0143)	(0.0148)
Gender (male)	−0.1363 ***	−0.0587 **	−0.0632 **	−0.2228 ***	−0.1307 ***	−0.1496 ***
	(0.0297)	(0.0299)	(0.0305)	(0.0327)	(0.0308)	(0.0322)
Age	0.0015	0.0040 ***	0.0033 ***	0.0011	0.0048 ***	0.0048 ***
	(0.0011)	(0.0011)	(0.0011)	(0.0012)	(0.0011)	(0.0012)
Education	−0.0185 ***	−0.0153 ***	−0.0180 ***	−0.0157 ***	−0.0168 ***	−0.0178 ***
	(0.0032)	(0.0033)	(0.0033)	(0.0035)	(0.0033)	(0.0035)
**Marital status (single as reference)**						
Married	−0.1195 ***	−0.1364 ***	−0.1056 **	−0.1630 ***	−0.3320 ***	−0.2104 ***
	(0.0417)	(0.0419)	(0.0430)	(0.0454)	(0.0420)	(0.0447)
Divorced	−0.2000 **	−0.1199	0.1354	−0.0469	0.0047	−0.1400
	(0.0988)	(0.0966)	(0.0935)	(0.1031)	(0.0929)	(0.1023)
Widowed	−0.1007	−0.0987	0.0061	−0.1602 *	0.2016 **	−0.0726
	(0.0873)	(0.0870)	(0.0880)	(0.0956)	(0.0832)	(0.0905)
**Religion (Western religions as reference)**						
Eastern	−0.0148	−0.1018	−0.1199	−0.0674	−0.0319	−0.0320
	(0.0805)	(0.0811)	(0.0814)	(0.0864)	(0.0829)	(0.0857)
No religion	−0.1119	−0.1207 *	−0.1889 ***	−0.1283 *	−0.1225*	−0.1113
	(0.0718)	(0.0719)	(0.0720)	(0.0766)	(0.0737)	(0.0759)
Income	−0.0203 ***	−0.0243 ***	−0.0246 ***	−0.0155 ***	−0.0198 ***	−0.0217 ***
	(0.0044)	(0.0045)	(0.0046)	(0.0049)	(0.0046)	(0.0048)
Working time	0.0011	−0.0008	0.0000	−0.0008	−0.0021	−0.0007
	(0.0013)	(0.0013)	(0.0013)	(0.0014)	(0.0014)	(0.0014)
Smoking (yes)	0.0034	0.0492	0.0472	−0.0302	0.0518	0.0330
	(0.0310)	(0.0310)	(0.0317)	(0.0350)	(0.0322)	(0.0339)
Drinking (yes)	0.0295	−0.0165	−0.0171	−0.0243	−0.0096	−0.0261
	(0.0297)	(0.0297)	(0.0305)	(0.0337)	(0.0310)	(0.0327)
Exercise (yes)	−0.0824 ***	−0.0934 ***	−0.1180 ***	−0.0432	−0.0257	−0.0792 ***
	(0.0263)	(0.0265)	(0.0272)	(0.0290)	(0.0272)	(0.0286)
Region	Yes	Yes	Yes	Yes	Yes	Yes
Pseudo-R2	0.0251	0.0205	0.0240	0.0326	0.0309	0.0304

Notes: Standard errors in parentheses; *** *p* < 0.01, ** *p* < 0.05, * *p* < 0.1.

**Table 4:** Influence of social support on mental health in different age groups (*N* = 14246).

Variable	Depression		Hopelessness		Failure		Fear		Loneliness		Meaninglessness	
	Age < 60	Age ≥ 60	Age < 60	Age ≥ 60	Age < 60	Age ≥ 60	Age < 60	Age ≥ 60	Age < 60	Age ≥ 60	Age < 60	Age ≥ 60
Friends	0.0004 **	0.0003	0.0000	−0.0005	0.0006 ***	−0.0004	0.0006 **	0.0006	0.0000	0.0004	−0.0002	−0.0000
	(0.0002)	(0.0008)	(0.0002)	(0.0009)	(0.0002)	(0.0009)	(0.0003)	(0.0009)	(0.0003)	(0.0008)	(0.0003)	(0.0008)
Speaking one’s mind	−0.0002	0.0012	0.0004	−0.0025	−0.0009	−0.0076 *	−0.0008	−0.0054	−0.0004	−0.0049	−0.0001	−0.0029
	(0.0004)	(0.0025)	(0.0004)	(0.0036)	(0.0007)	(0.0044)	(0.0007)	(0.0046)	(0.0010)	(0.0042)	(0.0010)	(0.0038)
Borrowing money	−0.0000	−0.0081 *	−0.0001	−0.0085 *	−0.0000	−0.0032	−0.0018	−0.0068	−0.0000	−0.0149 ***	0.0000	−0.0059
	(0.0002)	(0.0046)	(0.0002)	(0.0046)	(0.0002)	(0.0042)	(0.0015)	(0.0055)	(0.0002)	(0.0053)	(0.0002)	(0.0044)
Discussion	−0.0004	−0.0036	−0.0030	0.0027	−0.0033	0.0045	−0.0007	−0.0013	−0.0034	0.0005	−0.0011	0.0034
	(0.0008)	(0.0040)	(0.0020)	(0.0042)	(0.0022)	(0.0044)	(0.0013)	(0.0053)	(0.0025)	(0.0048)	(0.0017)	(0.0043)
Familiarity	−0.0639 ***	−0.0581 *	−0.0693 ***	−0.0055	−0.0812 ***	−0.0067	−0.0836 ***	−0.0627 *	−0.1037 ***	−0.0283	−0.0775 ***	−0.0489
	(0.0171)	(0.0308)	(0.0170)	(0.0312)	(0.0174)	(0.0316)	(0.0189)	(0.0350)	(0.0176)	(0.0322)	(0.0184)	(0.0330)
Trust	−0.0643 ***	−0.0642 *	−0.0789 ***	−0.0862 **	−0.0825 ***	−0.1013 ***	−0.0509 **	−0.0933 **	−0.0409 *	−0.1186 ***	−0.0711 ***	−0.1058 ***
	(0.0202)	(0.0353)	(0.0201)	(0.0353)	(0.0206)	(0.0358)	(0.0223)	(0.0398)	(0.0209)	(0.0364)	(0.0218)	(0.0375)
Mutual aid	0.0220	−0.0322	−0.0136	−0.0766 ***	−0.0156	−0.0620 **	0.0285	0.0509 *	−0.0345 **	−0.0254	−0.0428 **	−0.0714 **
	(0.0165)	(0.0264)	(0.0164)	(0.0261)	(0.0167)	(0.0264)	(0.0181)	(0.0304)	(0.0170)	(0.0272)	(0.0177)	(0.0280)
Control variables	Yes	Yes	Yes	Yes	Yes	Yes	Yes	Yes	Yes	Yes	Yes	Yes
Observations	10783	3463	10783	3463	10783	3463	10783	3463	10783	3463	10783	3463
Pseudo-R2	0.0239	0.0448	0.0208	0.0357	0.0266	0.0365	0.0315	0.0522	0.0290	0.0505	0.0299	0.0456

Notes: Standard errors in parentheses; *** *p* < 0.01, ** *p* < 0.05, * *p* < 0.1.
